# Selected Indonesian Medicinal Plants for the Management of Metabolic Syndrome: Molecular Basis and Recent Studies

**DOI:** 10.3389/fcvm.2020.00082

**Published:** 2020-05-06

**Authors:** Wawaimuli Arozal, Melva Louisa, Vivian Soetikno

**Affiliations:** Department of Pharmacology and Therapeutics, Faculty of Medicine, Universitas Indonesia, Jakarta, Indonesia

**Keywords:** curcumin, cinnamon, mangosteen, Indonesia, metabolic syndrome

## Abstract

Increased prevalence of metabolic syndrome (MetS) in the world influences quality of health in all respective countries, including Indonesia. Data from Indonesian Family Life Survey reported in 2019 showed that the prevalence of MetS in Indonesia currently is 21.66%, estimated with the provincial incidence ranging up to 50%; additionally, the most common components of MetS discovered in Indonesia were poor high-density lipoprotein (HDL) cholesterol and hypertension. Management treatment of MetS involves a combination of lifestyle changes and pharmacological interventions to decrease cerebrovascular disease. Various natural substances have been shown to govern any cardiovascular or metabolic disorders through different mechanisms, such as triggering anti-inflammation, lipid profile correction, sensitization of insulin reception, or blood glucose control. In Indonesia, the utilization of natural compounds is part of the nation's culture. The community widely uses them; even though in general, their effectiveness and safety have not been thoroughly assessed by rigorous clinical trials. Scientific evidence suggested that cinnamon, mangosteen, and curcumin, as well as their derived components possess a broad spectrum of pharmacological activity. In this review, an enormous potential of cinnamon, mangosteen, and curcumin, which originated and are commonly used in Indonesia, could be treated against MetS, such as diabetes, hyperlipidemia, hypertension, and obesity. The findings suggested that cinnamon, mangosteen, curcumin and their derivatives may reflect areas of promise in the management of MetS.

## Introduction

Metabolic syndrome (MetS) represents a group of physiological abnormalities involving central obesity, hypertension, dyslipidemia, and insulin resistance, which are strongly associated with an increased risk of cardiovascular event and adult-onset diabetes mellitus (T2DM) (1). Researchers reported that the prevalence worldwide of MetS was increased in both developed countries and developing countries, including Indonesia, one of the most populated countries in the region of Southeast Asian countries (2, 3).

Indonesia is a massive archipelago country with a projected population of 226 million in 2018 (Indonesia Population 2018), consisting of 33 provinces and various ethnic groups (estimated about 633), along with multiple cultures and lifestyles. A recent study from the Indonesian Family Life Survey, which is the first nationwide study regarding the prevalence and distribution of MetS in Indonesia, reported that its prevalence in Indonesia currently is 21.66%. The prevalence was conformed with the approximation of global MetS prevalence (20–25%) by the International Diabetes Federation (IDF) (IDF consensus worldwide 2006). The most common components of metabolic syndrome discovered in Indonesia were poor high-density lipoprotein (HDL) cholesterol and hypertension (3).

Along with modifying the underlying risk factors, the pharmacology therapy for MetS is aimed to treat the individual components of MetS (4). No single-drug therapy was available for MetS. The current pharmacotherapy is associated with treating comorbidities with chronic usage of multiple medications. Therefore, it is defying for patients to consume multiple drugs, leading to compliance reduction (5). Hence, the use of a natural compound in diminishing the risk of MetS is of interest, since the natural compounds seem to exhibit minimal side effects.

The results of a national survey in 2014–2015 showed a high prevalence of traditional medicines used in subjects with high blood sugar or heart problems, with about 9.9% used traditional medicines and 11.2% mixed modern and traditional medicines (6). About 5,000 species of medicinal plants can be found from the Medicinal Herb Index in Indonesia. Empirically, Indonesian herbal medicines are most commonly used as *Jamu*, a freshly prepared plant material, usually in the form of water extracts (7). Medicinal plants previously used for MetS in Indonesia, includes *Andrographis paniculata, Annona muricata, Zingiberis officinale, Cinnamomum burmanni, Garcinia mangostana*, and *Curcuma spp* (7).

Diverse natural compounds extracted from herbal plants, such as turmeric, garlic, cinnamon, ginger, grapes, onions, and broccoli exhibit verifiable benefits in the treatment of patients' MetS with the various mechanisms of actions (8). However, these natural compounds are still under investigation and are not recommended as a replacement for standard pharmacotherapies. In Indonesia, the utilization of natural compounds is part of the nation's culture. The community widely uses them; even though in general, their effectiveness and safety have not been thoroughly assessed by rigorous clinical trials. Indonesia has vast biological resources and is one of the most bio-diversified nations after Brazil (9).

Here, we review the most common existing natural compounds originating from Indonesia, which are cinnamon, curcumin, and mangosteen that have been studied for their benefits in managing MetS.

## General Description

### Cinnamon

Cinnamon is a common herb that is commonly applied in food and industries. Its oil could be used in traditional medicine as an antiseptic, antitussive, antimicrobial, antioxidant, or pain reliever (2).

In Indonesia, cinnamon was mainly found in Kerici, Sumatera, with the species called *Cinnamomum burmanni*. Availability of cinnamon for a long time in Indonesia, and it has become one of the primary commodities for trade since the Dutch colonization. The growth of *Cinnamomum burmannii* is optimally supported by the presence of mountainous land stretching along copious islands of Indonesia with enough rainfall. *Cinnamomum burmannii* is a shrub that is popularly recognized as the member of the *Lauraceae* family, such as Indonesian Cassia, Batavia Cassia, and Padang Cassia. It is widely distributed in Southeast Asia, cultivated in parts of Indonesia and Philippines possessing elliptical, green, anti parallelly arranged leaves and an ovoid-shaped fruit. The dried bark of the plant could be processed into rolls and quills for cooking. Cinnamon extract results in many chemical active compounds including flavonoids, phenols, alkaloids, quinones, steroids, saponins, and tannins (2, 10). Cinnamon's bark contains mainly procyanidins and catechins which are believed to result in pharmacological activity against type 2 diabetic and insulin resistance (11). In addition, cinnamon is also rich with essential oil mainly composed of eugenol and cinnamaldehyde which are known to be antimicrobial agents which exhibit a broad spectrum of activity (12).

### Mangosteen

Mangosteen (*Garcinia mangostana* Linn.) is an indigenous tree widely found in Southeast Asia, mainly Indonesia (13). Mangosteen fruit is round with thick skin (also called pericarp) and exhibits a purple color. Local people used various parts of *Garcinia mangostana* Linn. for treatment of diarrhea, wound infection, and fevers (14). Extensive pharmacological activities of mangosteen and its active compounds have been identified in many studies previously. The effects include anticancer, antibacterial, and antimalarial, as well as for the treatment of Alzheimer's disease (14, 15).

The main compound in mangosteen is xanthones. Up to date, more than 60 xanthones have been isolated from mangosteen fruit pericarp. Among the xanthones, alpha-mangostin (α-MG) and gamma-mangostin are the most prevalent. Other xanthones found include beta-mangostin, gartanin, mangostinone, isomangostin, and garcinones A, B, C, D, and E (16). Interestingly, a study by Muchtaridi et al. demonstrated high variability of concentrations of alpha-mangostin and gamma-mangostin extracted from *Garcinia mangostana* taken from various regions in Indonesia (17).

### Curcumin

Curcumin, which is mainly extracted from turmeric of the ginger family, is a polyphenol compound and used as a spice in Southeast Asian and East Asian countries, including Indonesia. Indonesia is a country with a wide diversity of *Curcuma* species, and it has been reported that Indonesia has 15 species of *Curcuma* in Java, among others are *C. aurantiaca, C. zedoaria, C. phaeocaulis, C. xanthorrhiza, C. longa* (18). Among the Curcuma species, *Curcuma longa* (turmeric) results in the highest amount of curcuminoids. The major curcuminoids discovered in the *Curcuma longa* are curcumin, bisdemethoxycurcumin, and demethoxycurcumin. It is poorly absorbed when given orally due to extensive first-pass hepatic metabolism to form major metabolites, like tetrahydrocurcumin and hexahydrocurcumin (19–21). To date, many studies demonstrated that curcumin can improve metabolic disorders. Several mechanisms have also been proposed to prove the mechanism of action of curcumin in association with these metabolic disorders. Ample evidence from *in vitro* and *in vivo* studies demonstrated that curcumin exhibits potent anti-inflammatory, antioxidant, anticancer, and antihyperglycemic activities (22, 23).

## Effects of Cinnamon, Mangosteen and Curcumin on Glucose Level and Diabetic-Related Outcome

### Cinnamon

Studies conducted by Cao et al. (24) examined the effects of *C. burmannii* extract and cinnamon polyphenols (CP) on the expression of the insulin receptor, glucose transporter 4 (GLUT4), and tristetraprolin (TTP/ZFP36) in adipocytes. Western blotting showed that CP increased IRβ levels, while both aqueous extract and CP elevated GLUT4 and TTP expression in the lipid tissues. Real-time PCR indicated that aqueous extracts (100 μg/mL) rapidly boost TTP mRNA by nearly 6-fold in the adipocytes. In fact, higher concentrations of *C. burmanii* decreased IRβ protein and mRNA quantity. Thus, the study proposed that the plant exhibits potential ability to enhance the process of metabolic transport and reactions, including insulin response, glucose transmembrane transport, and anti-inflammatory activity (24). Another study reported by Sheng et al. (25) concluded that cinnamon temporarily and competitively inhibits the alpha-glucosidase enzyme in improving symptoms of streptozotocin-induced diabetic rats. A study reported by Chen et al. (26) summarized that *C. cassia* and *C. tammala* extracts were rich in B and A type procyanidins oligomers, which are thought to be responsible for the antidiabetic activity of cinnamon. In this study, using diabetic (db/db) mice model which is a representative animal model for type 2 diabetic, they found that those 2 cinnamon extracts exhibit antidiabetic effects. Histopathological studies in pancreas, liver and adipose tissue showed that *C. cassia* promoted lipid accumulation in the liver and adipose tissue, whereas *C. tammala* improved the insulin concentration in the blood and pancreas (26).

A systematic review by Ranasinghe et al. (2) and Bandara et al. (27) on the effects of *Cinnamon zeylanicum* and *Cinnamon aromaticum* on diabetes mellitus, displayed a number of worthwhile effects both *in vitro* and *in vivo*, even though conflicting results were still under observation concerning supports from only few clinical trials. *In vitro, C. zeylanicum* reduced intestinal glucose assimilation by inhibiting activity of α-glucosidase and α-amylase. Furthermore, it stimulates cellular glucose uptake through direct expression of glucose transporter-4, inhibiting gluconeogenesis, and promoting insulin release, as well as insulin sensitization. The beneficial effects of *C. zeylanicum* in the body consist of diabetes-associated weight loss control, HDL cholesterol booster, reduction of fasting blood glucose, and increasing circulating insulin levels. *C. zeylanicum* also dramatically improved metabolic disorders related with insulin resistance and complication of diabetic neuropathy and nephropathy, without side effects of drug induced liver injury (2, 27).

Moreover, in a placebo-controlled double-blind clinical trial, 58 subjects of type 2 diabetic patients with HbA1c of more than 7% were treated with hypoglycemic agents plus 2 g of cinnamon or placebo daily for 12 weeks. At the end of the trial, HbA1c was significantly reduced in the cinnamon group (8.22–7.86%) as compared with the placebo group (8.55–8.68%). A reduction in mean systolic and diastolic blood pressure in the cinnamon-treated group (SBP = 132.6–129.2 mmHg; DBP = 85.2–80.2 mmHg) also occurred compared with the placebo group (SBP = 134.5–134.9 mmHg; DBP = 86.8–86.1 mmHg) (28).

A statistically significant subtraction of waist circumference, fasting plasma glucose, and body mass index was observed at month-3 compared to baseline in the cinnamon group; however, the changes were not different from the placebo with no significant differences shown in the total lipid profiles (29). Another clinical study reported the efficacy and safety of a novel bioactive substance (DLBS3233) extracted from *Lagerstroemia speciosa and Cinnamomum burmanii* in enhancing insulin sensitivity, as well as β-cell physiological function in patients with impaired glucose tolerance. DLBS3233 treated insulin resistance better than the placebo, represented through a lower value of HOMA-IR index. Restoration of the two-phase insulin secretion was constantly higher in the DLBS3233 group. Besides, DLBS3233 results in a much better oral disposition index than the placebo with no serious adverse events reported (30). Another double-blind, randomized, and placebo-controlled clinical trial reported the opposite result in which the type 2 diabetes patients were given either 3 g/day cinnamon supplement or a placebo. At the end of the intervention, alterations in the level of fasting blood glucose, insulin, HbA1c, HOMA-IR index, carboxymethyl lysine, total antioxidant capacity, and malondialdehyde were neither significant in both group nor between groups observed within these glycemic and inflammatory components (31).

### Mangosteen

Studies describing the hypoglycemic efficacy of mangosteen were mostly done in streptozotocin-induced diabetes rat model (32–35). Streptozotocin is considered the most post potent diabetogenic chemical used for diabetic research (36). Its toxic effects are dependent on its accumulation on pancreatic beta cells and produce damage via DNA fragmentation (37). However, due to the nature of various contributing factors in MetS, a streptozotocin-induced animal model is not an ideal model to study hyperglycemia in MetS. A previous review suggested that the rat model that displayed the closest criteria to human MetS was by inducing a high-carbohydrate and high-fat diet (38, 39).

Ethanolic extract of *Garcinia mangostana* was shown to exhibit a potent hypoglycemic effect in streptozotocin-induced diabetes in rats given for 28 days, which was related to the increase in beta cell number in pancreatic islet (32).

In streptozotocin-induced cell damage, alpha-mangostin promotes expression of both phosphorylated insulin receptor and pancreatic duodenal homeobox 1 (Pdx1), therefore, improving insulin secretion in the INS-1 cells. The effect of alpha-mangostin in INS-1 cells was followed by the increased phosphorylation of PI3K/Akt and ERK signaling pathways. As a result, the phosphorylation of insulin receptor substrate (IRS-1-Ser1101) was inhibited. Moreover, treatment with alpha-mangostin decreases the phosphorylation of c-Jun N-terminal kinase (JNK), p38 factor, and cleavages of caspase-3 in pancreatic β-cells (33).

Protective effects of alpha-mangostin in hyperglycemia-induced cell dysfunction were further explained by its capability to protect various cells from apoptotic damage (33, 40, 41). Alpha-mangostin was shown to exert its anti-apoptotic effect *in vitro*, by attenuating the effects of high glucose in human umbilical vein endothelial cells (HUVEC). The effects of high glucose shown in the study includes the up-regulation of cleaved caspase-3 and Bax, down-regulation of BCl-2, elevation of ceramide levels, and the increase of acid sphingomyelinase activity (29). Further, alpha-mangostin restored the high glucose-induced apoptosis regulated by the H19/miR-140/HE4 regulatory axis in HUVEC cells (42). In rat primary hepatocytes, alpha-mangostin increased mitochondrial membrane potential, decreased total ROS, and decreased mitochondrial ROS levels, followed by reduced Caspase-3 and Caspase-9 activities compared with the control group (41).

In cells cultured under high glucose conditions, similar evidence is found on aldose-reductase (AR)-dependent factor in ROS generation, confirming AR as the primary cause for the pathogenesis of numerous diabetic complications (43). Among xanthones extracted from *Garcinia mangostana*, gamma-mangostin followed by alpha-mangostin showed strong inhibitory properties on aldose reductase (44).

Ryu et al. (34) demonstrated that prenylated xanthones exhibit α-glucosidase inhibitory and antihyperglycemic activity in streptozotocin-induced diabetic rats, with gamma-mangostin being the most potent.

The activity of alpha-mangostin and gamma-mangostin in alleviating insulin resistance was explained in the primary cultures of human adipocytes. Alpha- and gamma-mangostin attenuated lipopolysaccharides (LPS) activation of the c-Jun NH(2)-terminal kinase, extracellular signal-related kinase, mitogen-activated protein kinases (MAPK), and activator protein (AP)-1 activity. Gamma- mangostin was shown to be more potent in attenuating the MAPK signaling pathway. Gamma-mangostin also controlled the adiponectin gene expression and insulin-stimulated glucose uptake and PPAR-γ (45).

Confirmation of long-term alpha-mangostin supplementation in type 2 diabetic rats was provided by Mekseepralard et al. (35) Alpha-mangostin given at 200 mg/kg BW/day for 40 weeks resulted in a decreased level of HbA1c, plasma cholesterol, fasting blood glucose, triglyceride, and followed by the increase serum insulin. Moreover, the long-term alpha-mangostin treated group demonstrated a better glucose tolerance pattern than the short-term-supplemented group (8 weeks).

Moreover, alpha-mangostin also is shown to improve micro hemodynamic indices in type 2 diabetic rats (46, 47). Alpha-mangostin improves micro hemodynamic mean arterial pressure, serum insulin, plasma HbA1c, blood cholesterol, triglyceride, and HOMA-IR index, compared with control diabetic rats (46). Further, α-MG treated DM2 rats restore ocular blood flow by decreasing retinal MDA, AGEs, RAGE, TNF-α, and VEGF (46). Interestingly, another study by the same group proves that α-MG is more effective than curcumin in improving MAP and retinal flow resistance in DM2 rats (37). Abdallah et al. (48) in his study, also confirmed that AGEs formation was inhibited by bioactive metabolites of *Garcinia mangostana*.

The protective effect of alpha-mangostin on diabetic nephropathy was reported in a diabetic rat model. Twelve-week after alpha-mangostin treatment, the diabetic kidney injury was reduced. The reduction of kidney injury was associated with a reduced level of acid sphingomyelinase and the down-regulation of ER stress-related protein expressions (49).

Mangosteen peel extract was also shown to exhibit a protective effect in the *in vitro* model of diabetic glomerulosclerosis, as the extract could promote cell growth in glucose-induced mesangial cells. It also significantly lowers the production of fibronectin and TGF-β1 (50).

Limited clinical evidence is available on the efficacy of mangosteen in diabetic patients. However, few clinical trials have been performed on healthy obese patients with the additional observation on fasting plasma glucose and HOMA-IR (51–53). Mangosteen supplementation was shown to improve insulin sensitivity in a pilot clinical trial of 22 obese female patients. HOMA-IR was significantly improved in patients treated with behavioral therapy plus mangostin (HOMA-IR: −53.22%) vs. behavioral therapy only (HOMA-IR: −15.23%), with no notable adverse effects (53).

### Curcumin

Curcumin is one of the natural products, among others, that received extensive attention for the prevention of progressivity of diabetic complications (54). Ample literature on curcumin shows its activity as an antidiabetic in animal models of diabetes with various doses and duration of research. Oral administration of multiple doses of curcumin, such as 80 mg/kg/day for 3 weeks (55) and 45 days (56); 60 mg/kg/day for 2 weeks (57); 100 mg/kg/day for 4 weeks, and 8 weeks (58) were able to prevent body weight loss, reduce blood glucose levels, and improve insulin sensitivity. Several possible mechanisms of the effects of curcumin in lowering blood glucose levels in diabetic animal models have been proposed, including the possibility that curcumin could attenuate plasma free fatty acids, tumor necrosis factor-α (TNF-α), nuclear factor-kappa B (NF-κB) activity, lipid peroxidation, and protein carbonyl. Also, curcumin decreased thiobarbituric acid reactive substances levels and the activity of sorbitol dehydrogenase (59, 60).

Recent studies showed that curcumin at a dose of 90 mg/kg/day plus insulin 1U/day vs. 4U/day given in streptozotocin-induced diabetic rats can decrease blood glucose levels, improve biomarkers of hepatorenal injury, attenuate lipid profile and hepatic antioxidants levels (61, 62). In addition, curcumin supplementation (0.02%, wt/wt) in db/db mice for 6 weeks improved homeostasis model assessment of insulin resistance (HOMA-IR), glucose tolerance, and significantly lowered blood glucose levels and HbA1c levels. The mechanisms that cause a positive effect on blood glucose levels is due to curcumin's ability to increase hepatic glucokinase activity and to decrease glucose-6-phosphatase and phosphoenolpyruvate carboxykinase activity (63). El-Moselhy et al. (64) reported that curcumin (80 mg/kg/day) administration for 75 days, obtained from *Curcuma longa*, in comparison with rosiglitazone (1 mg/kg/day) in high-fat diet-induced type 2 diabetes mellitus in rats exhibits an anti-hyperglycemic effect and also improved insulin sensitivity, and this action is due to at least in part by curcumin's and rosiglitazone's anti-inflammatory activity. In addition to the inflammation process, oxidative stress also demonstrates an important role in terms of its relationship to cause type 2 diabetes mellitus. A study conducted by Kaur and Meena (65) demonstrated that the combination of curcumin, piperine, and quercetin is a natural source of antioxidants that can prevent oxidative stress caused by high-fat feeding and low-dose streptozotocin injection, at least in part through increased antioxidant activity. Curcumin has also been known to be able to improve insulin resistance in skeletal muscle through increased oxidation of fatty acids and glucose which is likely mediated through the LKB1-AMPK pathway. Curcumin reduced plasma glucose and plasma lipid dose-dependently with a dose of 150 mg/kgBW showed the greatest effects compared with a dose of 50 mg/kgBW in high-fat diet and low-dose STZ injection-induced diabetes rats (66).

Up till now, the use of curcumin in humans has not been recommended in certain conditions, for example, in pregnant or nursing mothers, children, or in patients with anemia or liver disease due to few studies done with those conditions. Panahi et al. (67) demonstrated that curcuminoids 500–1,000 mg/day plus piperine 5–10 mg/day for 3 months decreased blood glucose levels significantly vs. a placebo in patients with type 2 diabetes. Also, curcuminoids also reduced C-peptide levels, HbA1c, TNF-α, and alanine and aspartate aminotransferase levels with no adverse effects (67, 68). Renal dysfunction is one of the serious complications of diabetes mellitus and is characterized by progressive decline of eGFR, albuminuria, and hypertension (69). Currently, diabetic nephropathy is the leading initial trigger of chronic kidney disease complicating in terms of mortality and morbidity for diabetic individuals. Ample evidence demonstrated the multiple mechanisms by which curcumin may attenuate renal damage due to diabetes. It has been shown that curcumin increases clearance of BUN and creatinine (58, 70, 71). Another study demonstrated that curcumin causes post-translational modification of H3 subtype histone and alteration of HSP-27 and p38 MAPK expression rate in kidney (70). Though curcumin could activate the p38-MAPK-HSP25 pathway in mouse podocyte, it failed to modulate the possibility of albuminuria in STZ-induced diabetic mice (72). Clinical trials then assured the effect of curcumin on end-stage renal disorders. They demonstrated that curcumin inhibits secretion of transforming growth factor- β (TGF-β), urinary protein, and interleukin (IL)-8 (73). A randomized, double-blind, and placebo-controlled trial conducted by Chuengsamarn et al. on two hundred forty patients with prediabetes proved that the administration of curcumin extract for 9-months improved the beta-cell function of pancreas, with lower HOMA-IR and higher adiponectin affected significantly as compared to the placebo (73). Na et al. (74) continued their study of curcumin for diabetes mellitus from experimental animals to trials in humans, which included one-hundred patients who were overweight/obese with type 2 diabetes mellitus, randomly divided into two groups, a curcumin supplemented (300 mg/day) group and a placebo group for a period of 3 months. In the study, they proved that administration of curcumin supplementation significantly reduced fasting plasma glucose levels, insulin resistance, HbA1c, serum triglycerides, and total free fatty acids as compared to the group receiving placebo (74).

## Effects of Cinnamon, Mangosteen, and Curcumin on Blood Pressure

### Cinnamon

An animal study by Nyadjeu et al. (75) evaluates the antihypertensive property of *Cinnamomum zeylanicum* extract in three animal models of hypertensive and in isolated rat aortic *in vitro* study. Acute intravenous injection of the extract caused a significant drop in mean arterial blood pressure in anesthetized four groups of rats, such as normotensive Wistar rats, salt-loaded hypertensive, L-NAME hypertensive, and spontaneously hypertensive rats. Pretreatment with either atropine or propranolol dramatically masked the therapeutic effects of the extract, deducing its probable mechanism in interfering both sympathetic and cholinergic transmissions (75).

Moreover, possible active vasodilatation was restrained in the pretreatment of L-NAME rats, which might be predominantly mediated by an endothelial nitric oxide synthase pathway. In an isolated mouse aortic study, they found that extract antihypertensive mechanism was partly due to vasodilation via endothelial nitric oxide and potassium ATP channel activation in vascular smooth muscle (75).

One systematic review from Mousavi et al. (76) discussed nine trials that enrolled a total of 641 subjects. Cinnamon induced drop in SBP (Weighted Mean Differences (WMD): −6.23 mmHg and DBP (WMD: −3.93 mmHg), which were optimally detected in trials using <2 g cinnamon, more than 12 weeks length of experimentation, and <50 years-old participants. However, no non-linear association was concluded between study duration and cinnamon supplementation dosage in the aforementioned systolic blood pressure value. Although cinnamon shows potential effects in controlling hypertension, further studies are necessary to be conducted in order to confirm its reviewed therapeutic effectiveness (76).

### Mangosteen

Healthy endothelium and blood vessels produce natural regulators of vascular tone and blood pressure. Endothelial cell dysfunction, as characterized by the impaired release of nitric oxide pathways, was found in hypertension (77).

A methanol extract of *Garcinia mangostana* fruit hulls showed a direct relaxant effect by increasing aortic nitric oxide in a MetS animal-isolated aorta (48). While in isolated rat aortic rings, γ-mangostin causes vasorelaxation via the NO-cGMP pathway. Also, γ-mangostin activates K^+^ channels and inhibits the influx of extracellular calcium ion, which supports the use of mangostin as an antihypertensive agent (78).

Up to date, no clinical trials have been performed in hypertensive patients. However, studies on healthy volunteers and obese patients showed that the use of mangosteen extracts resulted in minimum changes in the subjects' blood pressure (51, 53, 79).

### Curcumin

The increasing number of clinical trials proved that curcumin demonstrates cardioprotective efficacy, mainly due to its antihyperlipidemic and anti-atherosclerotic effects. A recent meta-analysis suggested that chronic supplementation of curcumin/turmeric could sustain a favorable impact on systolic blood pressure. Although the overall results did not show a dramatic decrease in systolic blood pressure and diastolic blood pressure after administration of curcumin, subgroup analysis showed that long-term administration of curcumin for more than 4 months was able to decrease systolic blood pressure but not diastolic blood pressure (80). These findings have been influenced by its low bioavailability during oral administration. More than half of the oral dose of curcumin will be excreted through feces, and only a small amount can be detected in plasma. This is because curcumin exhibits a poor solubility index, low intestinal permeability, and rapid first-pass biotransformation (81). The magnitude of reduction of systolic blood pressure was very slight following curcumin/turmeric supplementation; this is probably caused by curcumin, which can improve endothelial function. However, it has been proposed that the rate of ischemic heart disease and cerebrovascular accident were lowered by 7 and 10%, respectively (82). Yet, its possible mechanism is not well comprehended. Yao et al. (83) revealed the possible mechanism of reducing blood pressure by curcumin, curcumin decreased angiotensin type-1 receptor formation in concentration, and the time-dependent manner in the vascular muscle cells, as well as decreasing specificity protein 1 (SP1) binding with the angiotensin 1 receptor promoter. The positive effects of curcumin on L-NAME induced endothelial dysfunction are closely related with suppression of oxidative stress, increased endothelial NOS activity, and rapid regeneration of glutathione to balance the status of redox system (84). Another study by Xia et al. (85) showed that curcumin administration significantly lowered the blood pressure of male albino rats in time- and dose-dependent manner. The authors proved that curcumin significantly increased RBC velocity, micro vessel diameter, dynamic vasomotion, and open capillaries number. Hodai et al. demonstrated that patients with type 2 diabetes supplemented with triple-daily for 10 weeks with either curcumin (1,500 mg/day) or a placebo capsule showed no significant differences observed in the mean SBP and DBP within groups and between two groups. They also showed that there were no significant differences occurred in the body mass index between the two groups (86). Therefore, to provide curcumin as a blood pressure-lowering drug, further research is still required, especially in patients with hypertension and longer follow-up.

## Effects of Cinnamon, Mangosteen, and Curcumin on Lipid Profile

### Cinnamon

In a hypercholesterolemic zebrafish model, cinnamon was discovered to slow down the atherosclerosis process by preventing glycation of apoA-1 and inhibiting activity of the cholesteryl ester transfer protein (87). Further, in the atherosclerotic animal model induced by dexamethasone, treatment with cinnamon extract improved glucocorticoid-induced dyslipidemia compared to the dexamethasone control group (88). Also, the aqueous cinnamon reduced the expressions of scavenger receptor class A and T cell receptor CD36, as well as uptake of acetyl-low density lipoprotein via regulation of ERK1/2 activity in macrophages (89).

Besides, cinnamon triggers the formation of PPAR-α, PPAR-γ, and their target genes such as CD36 and GLUT4 membrane protein in adipocytes (25). Also, one of the active cinnamon ingredients, namely 2-methoxycinnamaldehyde, diminishes expression of vascular adhesion cell protein-1 in tumor necrosis factor-alpha (TNFα)-activated HUVECs (43). Furthermore, the cinnamophilin derived in cinnamon extract was found to be an antagonistic ligand for thromboxane A2 receptor, inhibiting any platelet aggregation (90). Taken together, these results highlight the beneficial effects of cinnamon in atherosclerosis. Up to date, no clinical trials have been performed in specific patients with dyslipidemia to explore the beneficial effect of cinnamon in lipid profiles.

### Mangosteen

Low-density lipoprotein (LDL) oxidation is an early event in atherogenesis. Therefore, the antioxidative effect of preventing oxidation of LDL has been the focus of atherosclerosis research (91). Alpha-mangostin acts as a radical species scavenger to keep the LDL from oxidative stress *in vitro* (92). Another study showed inhibition of α-tocopherol consumption induced by LDL-oxidation by alpha-mangostin (93).

A study was done by Shibata et al. (94) and they reported that crude mangosteen extracts given for 17-weeks could inhibit the development of atherosclerosis in ApoE–/– mice. In mice treated with crude mangosteen extracts, total cholesterol and triglycerides reduced significantly, followed by a decreased hepatic fatty acid transporter and 3-hydroxy-3-methyl-glutaryl-coenzyme A synthase (94). Ethanolic extract of mangosteen given at 800 mg/kg BW in rats had also been shown to improve lipid profile in high cholesterol diet, as well as decrease the level of hydrogen peroxide, the expression of NF-κB, inducible NOS pathway, HIF-1α, and maintain expression of endothelial NOS (95, 96). As a result, vasa vasorum angiogenesis, which occurs in atherosclerosis progression, also decreases (96).

In streptozotocin-induced rats, Taher et al. (32) showed that *Garcinia mangostana* extract did not only lower blood glucose levels but also reduced the levels of triglycerides (TG), LDL, total cholesterol (TC), very-low-density lipoprotein (VLDL), and increased HDL and total protein (TP).

In a small scale randomized controlled trial of 22 obese female patients, mangosteen extract 400 mg once daily for 26 weeks was reported to increase HDL cholesterol (58 mg/dL in the alpha-mangostin group vs. 49 mg/dL in control group). However, no improvement was found in other lipid profiles (TC, LDL-C, and triglycerides) (53). However, when *Garcinia mangostana* extract was administered in combination with *Sphaerantus indicus* flower heads extracts (also known as Meratrim) in clinical trials in overweight and obese subjects, significant reductions in TC (−13.8%) and triglycerides (−41.6%) were noted in the studies (53, 97).

### Curcumin

Hypertriglyceridemia triggered by a high intake of fructose-rich diets is a significant risk factor for cardiovascular disease. This may be caused by hepatic *de novo* lipogenesis, which then will increase the formation of free fatty acid rendering to hypertriglyceridemia. Excess triglycerides could be kept as lipid droplets in hepatocytes or secreted in the form of VLDL (98). The most common cause of liver damage that often coexists with MetS, such as diabetes and obesity, is the non-alcoholic liver disease. NAFLD often manifests as intracellular triglyceride accumulation complicating into inflammation (steatohepatitis), fibrosis, and eventually end-stage cirrhosis (99). If the simple steatosis is not treated early, it may progress to cirrhosis with ultimate failure of liver function and increases the risk for liver cancer pathogenesis.

Although vast ranges of lipid-lowering drugs are, dyslipidemia complications still exist. Curcumin has been studied for years for its benefits as an anti-inflammatory, antihyperlipidemic, and improved leptin resistance (43, 100, 101). Li et al. (22) reported that curcumin improves condition with hypertriglyceridemia and suppresses hepatic protein tyrosine phosphatase 1B formation to increase insulin sensitivity. A recent study has shown that curcumin could prevent hepatic steatosis and high-fructose-induced hyperlipidaemia in male Wistar rats via suppression of protein expression of LXR-α and SREBP-1c in the liver as well as decreases the production of lipogenic enzymes, such as acetyl-CoA carboxylase or fatty acid synthase (23). A meta-analysis of seven trials, including a total of 649 subjects, conducted by Qin et al. (102) showed that administration of curcumin and turmeric could protect patients against the risk of cardiovascular disease by improving serum lipid levels, that is curcumin and turmeric could significantly decrease serum LDL-cholesterol and triglyceride levels as compared to those in control group. Curcumin is utilized as dietary adjunct to standard drugs. However, further research is needed to resolve its uncertain dosage and medication frequency.

The mechanism behind the hypolipidemic activity of curcumin and turmeric is partly because curcumin can increase PPAR-γ, suppressing the expression of the LDL-C receptor, and thereby reducing plasma LDL-C levels (103, 104). A recent study was performed in which curcumin was administered at a dose of 200 mg/day and also a combination of phytosterols and curcumin at a dose of 2 g/day−200 mg/day, respectively, for 4 weeks to hypercholesterolemic individuals. The researchers found that the combination of phytosterols and curcumin supplementation could significantly lower TC, LDL-cholesterol, and ratio of total/HDL cholesterol as compared to that of single supplementation of phytosterol or curcumin. However, curcumin alone administration does not greatly reduce TC and LDL-cholesterol. They also stated that curcumin could potentiate the cholesterol-regulating effects toward phytosterol, suggestive for additive and synergistic effects (105).

## Effects of Cinnamon, Mangosteen, and Curcumin on Obesity

### Cinnamon

Kwan et al. (106) reported that cinnamon extract (CA) promoted multilocular brown fat phenotype in 3T3-L1 adipocytes. Moreover, it triggered expression of brown adipocytes markers but reduced expression of white adipocytes markers in the 3T3-L1 adipocytes. In this study, they also reported that cinnamon extract augmented brown adipocytes markers in the subcutaneous adipocytes isolated from diet-induced obesity (DIO) mice and db/db mice. In addition, the oral administration of the extract enhanced UCP1 expression in the subcutaneous adipose tissue, as well as body weight reduction in the DIO group.

Another *in vitro* study reported that CA rapidly increases protein kinase A signaling, rate of expression of several thermogenic genes, and HSL—PLIN1 phosphorylation in the murine primary adipocytes. Suppression of protein kinase A or p38 MAPK enzymatic activity dramatically down-regulated the CA-induced thermogenesis. Furthermore, long-term CA treatment modulates reprogramming of metabolic pathways partially diminished in FGF21KO subtype adipocytes lineage. As a matter of fact, both acute and chronic effects of extract administration in isolated human adipose stem cells were observed, derived from different ethnicities, ages, and body mass index (BMI). Meanwhile, CA triggers thermogenesis and heat metabolism in mouse and human subcutaneous adipocytes, providing a mechanistic explanation for the anti-obesity results which further supports its potential metabolic benefits (107). In addition, the impact of dietary polyphenols (which are known to be rich in cinnamon) on the gut microbiota and intestinal barrier functions were also tested. C57BL/6J mice were fed with a high-fat diet (HFD) for 8 weeks treated with extract from cinnamon bark. At the genus level, *Peptococcus* were decreased in HFD mice treated with cinnamon, and the expression of several antimicrobial peptides and tight junctions proteins such as ANG4 (effective against gram-positive and gram-negative bacteria, Reg3y (effective against gram-positive bacteria), and Lyz1 (mostly effective against gram-positive bacteria) was increased in response to cinnamon supplementation, indicating an improvement of gut barrier function (108).

A clinical study determines the impact of a 4-month treatment with dietary herbal supplements, such as chromium, cinnamon, and carnosine, toward either moderately obese or overweight pre-diabetic subjects. A 4-month treatment with the dietary supplement decreased FPG compared to placebo, without detectable significant changes in HbA1c. They did not find any changes in HOMA-IR markers, levels of serum insulin, lipid profile, and inflammatory markers among treatment groups. Although they did not find any alterations in weight and nutrient intakes between two groups, the proportion of fat mass diminished within the supplemented group compared to placebo one. A higher FPG concentration and milder inflammatory status benefited most from the supplementation (109).

In a 16-week double-blind randomized control trial, more than 100 individuals with MetS were divided into two groups, which were given cinnamon 3 g/day or wheat flour 2.5 g/day. Fasting plasma glucose, HbA1c level, waist circumference, and BMI was decreased in the cinnamon-treated group compared to the placebo one. Other parameters such as waist-hip ratio, total serum cholesterol, blood pressure, serum triglycerides, LDL cholesterol, and HDL cholesterol significantly improved (110). However, cinnamon did not change gastric emptying parameters, postprandial lipemia, glycemia, and appetite responses to high-fat breakfast in 9 healthy, young subjects supplemented with one dose of 3 grams powdered cinnamon. No significant effect of cinnamon treatment was observed on sensations of hunger, desire to eat and fullness (111).

### Mangosteen

Alpha-mangostin displayed its anti-obesity property *in vitro* by inducing apoptosis in 3T3 L1 (preadipocyte cells) as well as inhibiting fatty acid synthase at 20 μM (112). Moreover, alpha-mangostin was found to inhibit intracellular fat accumulation and prevent adipocytes maturation (112, 113).

Alpha-mangostin is highly distributed in the liver and fat *in vivo*, which makes it plausible to consider alpha-mangostin as anti-obesity (114, 115). Several mice or rat models of high-fat diet-induced obesity demonstrated the effect of mangostin in reducing body weight and retroperitoneal fat mass accumulation (41, 116, 117).

Chronic low-grade inflammation also contributed to adipogenesis (118, 119). Xanthones from mangosteen have been shown to prevent lipopolysaccharide (LPS)-mediated inflammatory response *in vitro* by modulating TNF-α, IL-6, interferon γ-inducible protein-10 (112). Chen et al. studied the anti-inflammatory mechanism of alpha- and gamma-mangostin in RAW 264.7 cells stimulated by lipopolysaccharide. The results showed that alpha- and gamma-mangostins significantly inhibited nitric oxide (NO) and PGE_2_. However, further analysis proved that the inhibitory activities of both xanthones are not due to direct inhibition of iNOS enzyme activity (120). A recent study proposed a mechanism by which alpha-mangostin successfully modulated LPS-stimulated IEC-6 cell inflammation using a genome-wide examination of control, LPS-stimulated, and alpha-mangostin-pretreated cells. The study showed that α-MG pretreatment reduced LPS-induced expressions of NLRP3, caspase 1, interleukin (IL)-18, and IL-1β (121).

Moreover, alpha-mangostin, and gamma-mangostin were able to prevent the activation of MAPK, NF-KB, and AP1, which can induce the transcription of pro-inflammatory cytokines released in obesity subjects (122). Shen et al. (123) found that alpha-mangostin exhibits potent activity in reducing NF-kB mediated inflammation and inhibiting Nrf2 promoter activity.

The previous study indicated antagonistic crosstalk between NF-κB and SIRT1 in the inflammatory signaling pathway. SIRT1 activation inhibits NF-κB signaling and suppresses inflammation. SIRT1 suppresses NF-κB signaling by deacetylating the p65 subunit of NF-κB complex (124–126). While in an *in vitro* study using the U937 cell line and human monocytes, alpha-mangostin was proven to inhibit p65 acetylation and down-regulate its downstream products, COX-2, iNOS via SIRT1 activation (127). In high-fat diet-induced obese mice, alpha-mangostin reduced body weight by upregulating hepatic AMPK, SIRT1, and PPAR-γ (117).

The effect of mangosteen juice on biomarkers of inflammation in 122 who were obese was evaluated after 8-week of supplementation, and the researchers found that in the highest dose evaluated (9 oz twice daily), improvements on hs-CRP and F2 isoprostane occurred. However, no significant changes in BMI and body fat percentages occurred (51).

The excessive oxidative stress in adipose tissues has also been shown to be linked with the pathogenesis of obesity (128). Mangosteen, given in the form of a single-dose functional beverage of *Garcinia mangostana* to healthy male and female subjects, increased plasma antioxidants and enhanced endogenous antioxidant activity (129).

Alteration in leptin expressions are also known to be important in obesity (130). Leptin is an adipocyte-secreted hormone that regulates appetite. An increase in leptin concentration will result in an anorexic effect (131). Yet, in wild type animals and in most human subjects, leptin concentrations increase by a HFD (132). *In vitro* study showed that alpha-mangostin increased mRNA expressions of leptin in 3T3-L1 adipocyte cells. However, Abuzaid et al. (133) reported that ethanolic extract of mangosteen pericarp significantly reduced resistin and leptin concentrations and reduced body weights in Wistar rats given HFDs.

Recent studies highlighted the role of gut microorganisms in the pathogenesis of obesity. Epidemiological studies in obesity subjects found reduced abundance in *Bacteroidetes* and an increase in *Firmicutes* phylum (134). Further, studies suggested that microbiome might stimulate reprogramming of gene expression in the gut (135, 136). Up to date, no study proved the efficacy of alpha-mangostin in improving dysbiosis in obesity. However, a recent study showed that alpha-mangostin altered intestinal microbiome, promoted dysbiosis and exacerbated colonic inflammation and injury in chemically-induced colitis in mice (137). Gutierrez-Orozco et al. (137) reported that alpha-mangostin significantly disrupts intestinal microbiomes, by reducing the amount of *Firmicutes* and increasing the abundance of *Proteobacteria*. The dysbiosis might be due to the inefficient absorption of alpha-mangostin that leads to high amounts of the compound transit in the colon and interacts with gut microbiota.

Clinical trials of mangosteen supplementation in subjects who are obese have been performed using several doses and in a variety of duration. A randomized controlled trial was conducted in 60 subjects who are obese with BMI between 30 and 40 kg/m^2^, receiving either herbal extract from *Spaeranthus indicus* and *Garcinia mangostana* or a placebo for 8 weeks. Herbal extracts resulted in a reduction in BMI of 1.61 kg/m^2^ as well as the reduction in waist circumference of 5.44 cm. An increase in adiponectin concentrations was also observed in the herbal group (52). In a randomized controlled trial of 22 subjects of obese female (BMI ≥ 30 kg/m^2^ with a bodyweight of <135 kg), mangosteen 400 mg once daily as a supplement to diet and physical activity for 26-week resulted in a weight loss of −4.5 kg in mangostin group vs. −1.42 kg in the placebo group. However, no significant difference was found regarding waist circumference and body composition vs. control (53).

*Garcinia mangostana* extracts given as herbal blend with extract of *Sphaeranthus indicus* (known as Meratrim) has been evaluated in several clinical trials of overweight and obese participants. In two identical clinical trials, 100 subjects with a BMI of 30–40 kg/m^2^ were given 400 mg of herbal blend twice daily or placebo for 8 weeks. At the end of the study, significant reductions in body weight (−5.2 kgs), BMI (−2.2 kg/m^2^), waist (−11.9 cm), and hip circumference (−6.3 cm) in herbal blend vs. the placebo occurred. The changes were followed by an increase in serum adiponectin concentration (52, 138).

The same combination was also studied in a placebo-controlled clinical trial in 60 overweight patients. Meratrim in 400 mg capsules was given to 60 participants with a BMI of 28.3 kg/m^2^ twice daily for 16 weeks. Large-scale body weight reduction (5.09 vs. 1.1 kg;), waist (9.97 vs. 3.71 cm), hip size (10.38 vs. 5.11 cm), and BMI (1.91 vs. 0.43 kg/m^2^) in the Meratrim group compared to the placebo one (97).

Taken together, anti-inflammatory and antioxidative properties of mangostin might be beneficial in modifying obesity and its related disorders.

### Curcumin

An ample of evidence suggests that specific dietary components found in spices, for instance, curcumin, may exert a beneficial effect on metabolism and possess anti-obesity and anti-inflammatory activities (139–141). Obesity is one of the risk factors of cardiovascular and kidney disease (142). Liu et al. (109) investigated the effects of curcumin on the prevention and treatment of obesity-related glomerulopathy. They explored its possible signal transduction pathway *in vitro* and *in vivo* experiments. They found that curcumin upregulated mRNA and protein expression of podocyte-associated molecules via leptin induction, like nephrin, podoplanin, podocins, and podocalyxin. They also proved that curcumin significantly reduced the body weight, Lee's index, abdominal fat index, urinary protein excretion, and average glomerular diameter and significantly upregulated the mRNA and protein expressions of the above podocyte-associated molecules in obesity-related glomerulopathy mice. The authors proved that the mechanisms of curcumin inhibit the obesity-related glomerulopathy was at least in part due to curcumin's ability to regress the high expression of Wnt2b, Wnt1, Wnt6, and β-catenin signaling on podocytes (109).

A recent study also demonstrated that supplementing HFD mice with curcumin and piperine could accelerate the caloric restriction-induced loss of total body fat. This combination is an effective intervention at suppressing high-fat diet-induced inflammation (143). A systematic review and meta-analysis of randomized controlled trials among patients with MetS and related disorders given by curcumin were assessed to find whether curcumin administration could reduce BMI and weight. A total of 1,163 studies were identified with total participants being 1,646 patients. The results of this systematic review and meta-analysis were that curcumin intake could be suggested as a useful supplement to be used for the management of the MetS. Curcumin intake significantly reduced BMI, weight, waist circumference, and increased adiponectin levels. However, the authors found a non-dramatic effect of curcumin intake on the hip ratio (144).

Curcumin is a polyphenol with an aromatic ring and one or more hydroxyl groups. It is now known that polyphenols exhibit biological effects following chemical modification carried out by gut microbiota. Actually, enzymes released by gut microbiota will perform deglycosylation, dehydroxylation, and a demethylation of polyphenols resulting in small catabolic products, which are easily absorbed during intestinal transit (145). Recently, several enteric bacteria have been identified that are capable of modifying curcumin, namely *Escherichia coli, Blautia* spp., *Escherichia fergusonii, Bifidobacteria longum, Bifidobacteria pseudocatenulaum, Lactobacillus acidophilus*, and *Lactobacillus casei*. All of these gut microbiotas can metabolize curcumin into two derivatives, demethylcurcumin and bisdemethylcurcumin (146). In fact, after oral administration of curcumin and metabolism of curcumin in the liver, curcumin in the intestine will change the ratio between pathogenic bacteria and beneficial bacteria by increasing the abundance of *Bifidobacteria* and *Lactobacilli* and reducing the loads of *Prevotellaceae, Coriobacterales, Enterobacteria*, and *Enterococci* (147).

Gut dysbiosis is defined as an alteration in the composition of gut microbiota and this alteration can occur due to exposure of various environmental factors including food, drugs such as antimicrobial agents, toxins, pathogens, and increased stress (148). Several studies have shown that a tight interaction exists between the gut microbiota dysbiosis and diabetes mellitus and obesity (149, 150). The alteration of composition of gut microbiota can also be induced by consumption of high-fat food, at least in part by increasing pathogen microbiota such as *Rikenellaceae* and decreasing *Ruminococcaceae* (151). Shen et al. (152) demonstrated that the administration of curcumin by gavage at a dose of 100 mg/kgBW for 15 days in C57BL/6 mice significantly decreased the abundance of *Prevotellaceae* while increased the abundance of *Bacteroidaceae* and *Rikenellaceae*. *Prevotella* species is a gram-negative bacteria that is very commonly found in the gut of patients with colorectal cancer (153). Moreover, Bereswill et al. (154) also demonstrated the beneficial effects of resveratrol, curcumin, and simvastatin in a murine model of hyper-acute Th1-type ileitis following orally infected by Toxoplasma gondii. Resveratrol, curcumin, and simvastatin equally could ameliorate acute small intestine inflammation by upregulating anti-inflammatory cytokines, IL-10 in ileum, mesenteric lymph nodes, and spleen and also increase the abundance of anti-inflammatory lactobacilli and bifidobacteria. All of the three compounds could maintain intestinal barrier function.

## Future Directions

In summary, a considerable amount of studies was carried out on the effects of cinnamon, mangosteen, and curcumin on MetS. The similarity of the results of the three extracts on MetS was based on their antioxidant, anti-inflammatory, and anti-apoptosis effects.

Overall, based on *in vitro* and *in vivo* studies, all three extracts, cinnamon, mangosteen, and curcumin exhibit excellent organ-targeted effects on MetS, as summarized in Table 1. While based on the available data on clinical trials that have been conducted, the studies were performed only on selected components of MetS such as hyperglycemia, diabetes, dyslipidemia, or obesity.

**Table 1:** Summary of pharmacological activity of cinnamon, mangosteen, and curcumin in target organ.

**Plant name**	**Extract/compound used**	**Cells/organ studied**	**Pharmacological activity**	**References**
**Cinnamon**				
Cinnamon zeylanicum	Methanol extracts of bark	intestine	Competitive, reversible inhibition on α-glucosidase enzyme (Wistar rats)	(155)
Cinnamon zeylanicum	Cinnamaldehyde	Skeletal muscle Liver tissue	Restore GLUT4 protein content Improvement in altered enzyme activities of pyruvate kinase and phosphoenolpyruvate carboxykinase (Wistar rats)	(156)
Cinnamon cassia	Ethanol extract of bark	Adipose tissue and liver	Promote lipid accumulation (db/db mice)	(26)
Cinnamon tamala	Ethanol extract of bark	Blood and pancreas	Improved insulin concentration (db/db mice)	(26)
Cinnamon cassia	Water extract	Adipocyte cell line	Increased protein level of PPAR–γ (3T3-L1)	(25)
Cinnamon zeylanicum	Water extracts	*In vitro* (BSA-Glucose assay)	Inhibit the formation of advanced glycation end-products	(157)
**Mangosteen**				
Garcinia mangostana	Ethanolic extract	Plasma	↓ Blood glucose (Wistar rats) ↓ Total cholesterol and hydrogen peroxide (Wistar rats, Sprague Dawley rats, ApoE deficient mice)	(32, 94–96)
	Alpha mangostin	Plasma	↓ HbA1c, Total cholesterol, LDL, TG, VLDL, fasting blood glucose (Sprague Dawley rats) ↑ HDL and total protein (Sprague Dawley rats) ↑ Insulin, ↓ HOMA IR (Sprague Dawley rats) ↓ Hs-CRP and F2-isoprostane ↑ Adiponectin (in human volunteers) ↑ Leptin (Wistar rats)	(32, 35, 46, 47, 51–53, 97, 133)
	Alpha mangostin		Negligible effect of blood pressure (obese female patients, albino mice)	(53–55)
	Alpha-mangostin	Pancreatic islet	↑Σ of beta cells (Sprague Dawley rats) ↑ Expression of insulin receptor and Pdx1 (INS-1 cells) ↑ Insulin secretion (Sprague Dawley rats) ↑ Phosphorylation of PI3K/Akt & ERK (INS-1 cells) ↓ Phosphorylation of JNK (INS-1 cells)	(32, 33)
	Alpha mangostin	Endothelial cells	Anti-apoptotic (INS-1 cells, HUVEC cells, Sprague Dawley rats) Regulates H19/miR/HE4 (HUVEC cells)	(33, 40–42)
	Alpha mangostin	Hepatocytes	↑ Mitochondrial membrane potential (Sprague Dawley rats) ↓ Total ROS and mitochondrial ROS (Sprague Dawley rats) ↓ Caspase-3 and Caspase-9 (Sprague Dawley rats) ↓ Hepatic fatty acid transporter (ApoE deficient mice) ↓ HMG-CoA synthase (ApoE deficient mice) ↓ NF-KB, iNOS, HIF-1α (Wistar rats)	(41, 94–96)
	Alpha- and gamma- mangostin	Adipocytes	↓ JNK, MAPK, AP1 ↑ PPAR-γ (primary culture of human adipocyte) ↑ Apoptosis in preadipocytes cells Inhibit fatty acid synthase (3T3-L1) ↓ NLRP3, Caspase 1, IL-18, IL-1β (3T3-L1 cells, primary culture of human adipocytes) ↓ NF-kB (primary culture of human adipocytes) Inhibiting Nrf2 activity (3T3-L1 cells) ↑ AMPK, SIRT1, PPAR–γ (C57BL mice) ↑ Leptin (3T3-L1 cells)	(45, 112, 113, 117, 121, 123, 132)
	Alpha- mangostin	Retina	↓ MDA, AGEs, RAGE, TNF-α, VEGF (Sprague Dawley rats) ↓ Retinal flow resistance (Sprague Dawley rats)	(46, 47)
	Alpha- mangostin	Mesangial cells	↓ Fibronectin (SV40 MES 13 cells) ↓ TGF-β1 (SV40 MES 13 cells)	(50)
	Alpha-mangostin	Aorta	↑ NO (Wistar rats) Vasorelaxation via NO-cGMP pathway (Wistar rats)	(78)
	Gamma- mangostin	Aorta	Activates K^+^ channels (Wistar rats) Inhibits influx of Ca^2+^ (Wistar rats)	(78)
	Alpha-mangostin	Gut	↑ Gut dysbiosis (C3H, Balb/c, Nude FoxN1nu and C57BL/6J mice)	(137)
Turmeric	Extract	Plasma	Reduced blood glucose, Hb, and glycosylated hemoglobin levels (Alloxan-induced Albino rats)	(55)
Curcumin	Curcumin compound	Cerebellum	Increase gene expression of Ach, Glut3, Muscarinic M1, M3, alpha7 nicotinic acetylcholine, & insulin receptor (Streptozotocin-induced male Wistar rats)	(57)
Curcumin	Curcumin compound	Kidneys	Increase creatinine clearance, reduce blood urea nitrogen, activities of PKC-α, and PKC-β1 and phosphorylated of ERK1/2 (streptozotocin-induced male Sprague Dawley rats)	(58)
Curcumin	Curcumin-supplemented yogurt	Adipose tissue, muscular tissue, plasma, urine	Reduce urine urea & glucose, proteinuria, serum triacylglycerol, & activities of aspartate & alanine aminotransferases. Increase hepatic glycogen (streptozotocin-induced diabetic rats)	(62)
Turmeric	Curcumin	Kidney	Down-regulate mRNA & protein expression of Wnt1, Wnt2b, Wnt6, & Beta catenin & up-regulate phosphorylation of beta-catenin protein in podocytes & renal tissues (immortalized mouse podocyte cell line & high-fat diet-induced C57BL/6J mice)	(109)
Turmeric	Curcumin	Adipose tissue and plasma	Decrease IL-1β, IL-6 Increase loss of body fat high-fat diet-induced C57BL/6J mice)	(143)
Curcumin	Curcumin	Small intestine	Increase IL-10, lactobacilli, bifidobacteria Decrease IL-23p 19, IFN-γ, TNF-α, IL-6, MCP-1 (C57BL/10ScSn mice)	(154)
Curcumin	Combination extract of curcumin: piperine: quercetin in a ratio 94: 1: 5)	Skeletal muscle	Increase AMPK, CD36, carnitine palmitoyltransferase 1 Decrease pyruvate dehydrogenase 4, phosphorylated glycogen synthase (High-fat diet and low-dose streptozotocin-induced albino female wistar rats)	(66)

*↑ Increased; ↓ Decreased*.

We also note that the three compounds show variable outcomes in different components of MetS. Cinnamon demonstrated better outcomes in hypertension, while mangosteen in obesity and curcumin on patients with hyperglycemia.

In order to be certain on the efficacy and safety of the three medicinal plants, well-designed clinical trials, with representative sample sizes, are still needed. Up to date, results from clinical trial data available are inconsistent, not to mention the limited number of studies are available on patients with MetS. Further, the number of patients recruited was also confined. We also note the heterogeneity in the results of clinical trials. The results' heterogeneity might be due to the various sources of the extracts which leads to the non-uniformity of ingredients used in the studies. The level of purity in the active compounds used also varied. Previous studies confirmed several findings of heavy metals or pesticides contaminations from contaminated soil or production process (158).

Limited bioavailability is among the challenges which make dose findings in human trials difficult. Unlike active compounds of cinnamon that have relatively good bioavailability, mangosteen and curcumin are poorly absorbed (16, 159, 160). Moreover, animal studies revealed that limited bioavailability of alpha-mangostin might lead to high amounts of compound in the gut, which resulted in the dysregulation of gut microbiomes (137, 161). Studies revealed that the interactions between gut microbiota and herbal compounds can be attributed to absorbable active small molecules and changed gut microbiota and its secretion (162), but at present, no studies informed the role of cinnamon, curcumin and mangosteen in this issue.

Limited and conflicting data from clinical trials in MetS, non-uniformity of quality and sources, and limited long-term safety results, among other things, are still preventing recommendations of these compounds for use in MetS.

Based on the current review, we saw promising results with a clear mechanism of actions of cinnamon, mangosteen, and curcumin. To confirm their efficacy in patients, further well-designed clinical studies need to be done to explore the effects on standardized effects with a large number of MetS patients. Further, long-term human studies are also needed, considering the possibility of long-term use of the drugs in MetS.

## Author Contributions

WA, ML, and VS contributed manuscript writing. WA revised and finalized the manuscript.

## Conflict of Interest

The authors declare that the research was conducted in the absence of any commercial or financial relationships that could be construed as a potential conflict of interest.
